# Editorial: Enhancing livestock production and food safety through a One Health approach in resource poor settings

**DOI:** 10.3389/fvets.2022.1079463

**Published:** 2022-12-13

**Authors:** Bassirou Bonfoh, Barbara Wieland, Hung Nguyen-Viet, Katharina Kreppel

**Affiliations:** ^1^Centre Suisse de Recherches Scientifiques en Côte d'Ivoire, Abidjan, Côte d'Ivoire; ^2^Institute of Virology and Immunology, Mittelhäusern, Switzerland; ^3^Department of Infectious Diseases and Pathobiology, Vetsuisse Faculty, University of Bern, Bern, Switzerland; ^4^International Livestock Research Institute, Hanoi, Vietnam; ^5^International Livestock Research Institute, Nairobi, Kenya; ^6^School of Life Sciences and Bioengineering, Nelson Mandela African Institution of Science and Technology, Arusha, Tanzania; ^7^Department of Public Health, Institute of Tropical Medicine, Antwerp, Belgium

**Keywords:** One Health, animal source food, systems approach, transformational knowledge, added value

This Research Topic aimed to collate scientific studies that demonstrate the theoretical foundation and operationalization of One Health considering the animal source food systems and livelihoods. We therefore paid attention to select studies that, from the start, applied system thinking and transdisciplinarity approaches, and tried to frame the food system problem at hand as part of a bigger system with discussions that addressed socio-ecological complexities, challenges and solutions based on clear engagement and equity. In the submissions we particularly looked for evidence of the One Health indicators: (i) collaboration, (ii) added value, (iii) system thinking, (iv) transdiciplinarity, (v) participation of stakeholders, (vi) gender and equity, (vii) implementation of action based on findings, (viii) sustainability.

Following a One Health approach requires transdisciplinarity and participation of different stakeholders (1, 2). An important challenge however, is to ensure that everybody understands the same thing. To explore how participants in a study on antimicrobial use and resistance in Uganda and Kenya understand questions in a survey, and to find ways to restructure and clarify the survey, Wenemark et al. present cognitive interviews as a promising method. Their approach helps to validate a questionnaire, and thus improve the quality of a survey. In particular for complex research questions following a One Health approach, this type of survey validation in our view is recommended.

Focusing on stakeholder participation, Ngwili et al. used focus group discussions in Uganda with different stakeholder groups along the pork value chain, combined with key informant interviews. Their findings demonstrate fragmented knowledge on the zoonotic parasite *T. solium* in different stakeholder groups, which in turns helps to devise content of stakeholder specific intervention programs. Asakura et al.'s work in Tanzania provide another example of how participatory approaches further illuminate complex problems and help to find a way forward. They added insights using participatory rural appraisals to a previous body of knowledge on brucellosis control in Tanzania, which was derived with quantitative tools, and with this expect to design more sustainable and acceptable community-based disease control programs. Similarly, by using stakeholder participation, Kemp et al. provide insight into common practices and awareness of farmers and veterinary professionals of antimicrobial use and antimicrobial resistance in Kenya. The study suggests sustaining several behavioral interventions in tandem with legislative reforms could reduce inappropriate prescription.

The advantages of combining qualitative and quantitative approaches were shown by Adjei et al., when assessing food safety challenges in the beef value chain in Ghana. Not only included the study several pathogens, but their occurrence could be linked to knowledge on food safety among butchers and retailers.

The importance of considering the “added value” is illustrated by Soare et al. Any intervention leads to some change in a system, ideally leading to benefits beyond the initially targeted areas. The authors thus argue, that pre-identifying potential synergies and trade-offs in disease control interventions is important during the design stage.

Lam et al. provide a rare example of how One Health thinking is applied already at the conceptual stage of a project in Vietnam. They integrate One Health in a Theory of Change framework to help characterize the pathways to safer pork in Vietnam.

Knowledge of the extent of a problem is not sufficient to find sustainable solutions; a fact that is presented by Davis et al. Based on findings from focus groups discussions in Tanzania, they report a range of animal health seeking strategies of livestock owners and identified access to resources and trust in health care providers as important factors influencing the ability of livestock farmers to act to improve livestock health.

System thinking by collecting evidence for policy is the approach chosen by Haile et al. The prevalence of *E. coli* in raw beef is determined across Ethiopia's capital and the resistance to antimicrobials is established.

Seko et al.'s interdisciplinary study applies quality theory based on an information economics approach to the user oriented quality perception of braised (dibiterie) meat in Dakar, Senegal. The study finds that consumer decisions if and where to buy braised meat, are based on subjective preferences and are not linked to food safety.

The One Health basic principles found in most studies, were transdisciplinarity and system thinking, followed by implementation of findings and stakeholder participation. Sustainability was found in only one study, while the indicators gender and equity were completely absent. Encouragingly, most studies aim to implement “better action”, but are missing examples of studies that show this process. This in turn means a lack of examples that demonstrate the “added value” of using a One Health approach even though its importance is stressed by Soare et al. This collection of papers features good examples of interdisciplinarity, but reaching true transdisciplinarity seems more of a challenge. Most studies focused on participation of different stakeholders, which is a positive development and has led to new insights on how challenges at the animal and human health interface can be addressed, that may indicate a positive trend toward system thinking.

We further observed that authors struggled to tease out the added value of collaborative work resulting from the One Health approach. A likely reason could be, that at the design stage of the studies, classical epidemiological principles are used and the One Health focus is an add-on at a later stage. It should be the other way round. The complexity at hand should be initially looked at from a One Health perspective followed by “zooming in” on a particular research question around collaboration and impact. With such an approach, it is more likely that factors linked to a particular problem are comprehensively considered allowing the discussion of the results within the system and not as stand-alone findings. The ownership of the produced co-designed transformational knowledge should then ideally lead to cost-effective and sustainable interventions in food safety.

Overall, it becomes clear that an adapted and improved Research Topic as follow up to this special edition is justified to provide a platform for One Health research and its implementation, incorporating the One Health principle from the onset. The study design should clearly show the process of identifying the problem and the One Health framework used to shape the research or intervention and the validation of findings involving all actors. While the One Health approach is gaining more traction, researchers in food safety are still finding their feet on how to present such work. Likewise, researchers claiming to use the One Health approach still need to develop their skills further. A future issue should thus center on practical cases and best practice to facilitate learning, while focusing on factors of success and failures in operationalizing One Health in food systems.

## Author contributions

BB and KK developed the first draft. BB, BW, HN-V, and KK revised the draft and approved the final version. All authors developed the first structure. All authors contributed to the article and approved the submitted version.
